# TiO_2_ nanowire-templated hierarchical nanowire network as water-repelling coating

**DOI:** 10.1098/rsos.171431

**Published:** 2017-12-20

**Authors:** Tian Hang, Hui-Jiuan Chen, Shuai Xiao, Chengduan Yang, Meiwan Chen, Jun Tao, Han-ping Shieh, Bo-ru Yang, Chuan Liu, Xi Xie

**Affiliations:** 1 State Key Laboratory of Optoelectronic Materials and Technologies, School of Electronics and Information Technology; The First Affiliated Hospital of Sun Yat-sen University; Guangdong Province Key Laboratory of Display Material and Technology, Sun Yat-sen University, Guangzhou, China; 2 Institute of Chinese Medical Sciences, University of Macau, Avenida da Universidade, Taipa, Macao SAR, China; 3 Department of Photonics and Display Institute, National Chiao Tung University, Hsinchu, Taiwan, Republic of China

**Keywords:** micro-nanoscale structure, hierarchical nanowires, self-cleaning surfaces, superhydrophobic coatings, nanowire thin film

## Abstract

Extraordinary water-repelling properties of superhydrophobic surfaces make them novel candidates for a great variety of potential applications. A general approach to achieve superhydrophobicity requires low-energy coating on the surface and roughness on nano- and micrometre scale. However, typical construction of superhydrophobic surfaces with micro-nano structure through top-down fabrication is restricted by sophisticated fabrication techniques and limited choices of substrate materials. Micro-nanoscale topographies templated by conventional microparticles through surface coating may produce large variations in roughness and uncontrollable defects, resulting in poorly controlled surface morphology and wettability. In this work, micro-nanoscale hierarchical nanowire network was fabricated to construct self-cleaning coating using one-dimensional TiO_2_ nanowires as microscale templates. Hierarchical structure with homogeneous morphology was achieved by branching ZnO nanowires on the TiO_2_ nanowire backbones through hydrothermal reaction. The hierarchical nanowire network displayed homogeneous micro/nano-topography, in contrast to hierarchical structure templated by traditional microparticles. This hierarchical nanowire network film exhibited high repellency to both water and cell culture medium after functionalization with fluorinated organic molecules. The hierarchical structure templated by TiO_2_ nanowire coating significantly increased the surface superhydrophobicity compared to vertical ZnO nanowires with nanotopography alone. Our results demonstrated a promising strategy of using nanowires as microscale templates for the rational design of hierarchical coatings with desired superhydrophobicity that can also be applied to various substrate materials.

## 1. Introduction

Self-cleaning surfaces, which possess extraordinary water repellency properties are currently the focus of considerable research [1–3]. They can be applied to the fields including fabric coatings [4,5], anti-biofouling paints [6,7], liquids separation [8,9], self-healing surfaces [10,11] and microfluidic devices [12,13]. One of the most well-known examples of self-cleaning surfaces in nature is the lotus leaf that can effortlessly roll off water drops. The underlying micro-nano hierarchical structure of lotus leaf surface has been revealed to play a key role in the water-repelling properties [14], and a large number of novel self-cleaning surfaces have been inspired by the micro-nano hierarchical structure of lotus leaf surface [1,2,15]. To understand the wetting behaviours of hierarchical surface, the Wenzel and Cassie–Baxter models are generally considered. In the Wenzel state, liquid impregnates the textures on solid surface, and the wetting or non-wetting feature of the solid is amplified due to the increase of the contact area between the liquid and the solid substrate [16]. While in the Cassie–Baxter state, air is trapped between the solid–liquid interface, which stably supports liquid drops sitting on the top of the substrate surface [17]. According to the Cassie–Baxter model, the presence of air pockets provided by the micro-nano hierarchical structure is crucial for superhydrophobic property. So far, it is commonly accepted that both hierarchical micro-nano structure and low surface energy coating on the solid surface are significantly important in achieving superhydrophobic effects. While low surface energy is generally prepared through surface functionalization with highly hydrophobic organic compounds, surface topography of micro-nano structure requires sophisticated fabrication or coating techniques.

To date, most micro-nanoscale structures have been pre-fabricated on substrates by either top-down or bottom-up approaches [13,18–23]. The micro-nanoscale structures were physically attached to the substrate after fabrication, and were further functionalized to be superhydrophobic, forming a robust surface with water-repelling effects. For example, hierarchical Al, Si, ZnO, SiO_2_ based surfaces with various surface roughness or surface topographies have been reported with excellent wetting properties [19,24–27]. However, the pre-fabrication process of micro-nanoscale structures and the requirement of physical attachment limit the choices of substrate material. Such approach relies heavily on the intrinsic properties of the used substrate, and can become unsuitable especially for the cases when the substrate material is unable to be fabricated with sub-micro features, or when the substrate material is susceptible or vulnerable to the fabrication process.

On the other hand, superhydrophobic surface produced by coating or drop-casting micro-nanoscale objects on substrate rather than through direct fabrication may get rid of the restriction of substrate types [28]. In such approaches, the micro-nanoscale objects were physically absorbed or coated on the substrate, which allows a variety of substrates to be functionalized with superhydrophobicity regardless of the intrinsic material of substrate. For example, superhydrophobic coatings were fabricated by self-assembling micro- and nano-sized silica spheres [29,30]. Superhydrophobic and superamphiphobic structures were also designed using candle soot as the template [31–33]. Generally the micro-to-nanoscale characteristics in these structures were produced by using microspheres or micropowders as underlying micro-scale templates, followed by decorating with nano-scale objects such as nanoparticles or nanowires (NWs) on the micro-scale templates. However, due to the spherical nature, microparticles have limited contact area with substrate, exhibiting low adhesion with substrate so that they are improper templates for constructing a robust superhydrophobic coating. In addition, when microparticles are drop-casted on the substrate, it is difficult to maintain the surface uniformity. The produced rough surface by stacking microparticles usually has large variations in roughness and uncontrollable defects. As a result, the surface morphology and wettability of the resultant micro-nanoscale structures cannot be well controlled.

Recently, much research has been focused on investigating the wetting behaviours of metal oxide semiconductors such as TiO_2_ and ZnO for desirable functional properties [28,33–37]. For instance, Lu *et al*. created an ethanolic suspension of perfluorosilane-coated dual-scale TiO_2_ nanoparticles as an excellent water-repellent paint [34]. Campbell *et al*. studied the electrowetting properties of ZnO nanorods with sputtered Teflon. Reversible wettabilities were observed under a threshold voltage of 35 V [35]. Sun *et al*. synthesized ZnO hollow microspheres with robust superhydrophobicity [28]. Although there has been significant progress in fabricating hydrophobic coatings, the possibility of using nanowires as building blocks for superhydrophobic hierarchical structure assembling is worth exploring. In this work, one-dimensional (1D) TiO_2_ NWs were employed as microscale templates for the fabrication of micro-nanoscale hierarchical nanowire network to construct superhydrophobic coating. The TiO_2_ NWs were coated on substrate by drop-casting method followed by sputtering with a thin ZnO layer. Due to their one-dimensional feature, the TiO_2_ NWs of micrometres length formed a robust network film, with each NW sufficiently contacting with the substrate or entangling with other NWs. Secondary ZnO NWs were branched on the TiO_2_ nanowire backbones through hydrothermal reaction, which produced nanoscale topography on the TiO_2_ NW templates, forming a hierarchical coating of NW network on substrate with homogeneous morphology. The hierarchical surface was further functionalized with fluorinated organic molecules, and exhibited excellent repelling properties against both water and cell culture medium in contrast to flat substrate or vertical ZnO NW substrate. Contrary to the conventional pre-fabrication techniques where substrate materials were limited, our work demonstrated a promising and universal strategy to produce homogeneous micro-nano textured superhydrophobic coating on solid surface that may be applicable for a wide range of substrate materials.

## 2. Results

**Figure 1.**
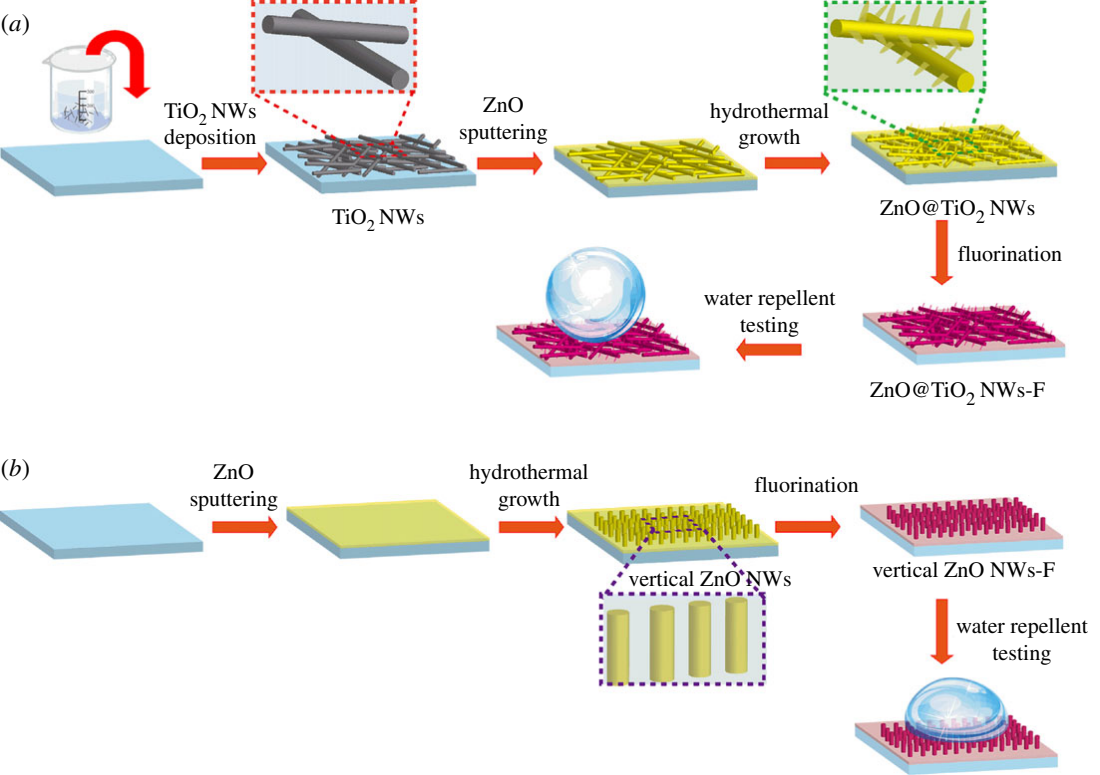
Schematic of the fabrication and the illustration of water-repelling properties of (*a*) hierarchical ZnO@TiO_2_ NW coating and (*b*) vertical ZnO NW film.

The schematics shown in figure 1 depict the fabrication procedure of hierarchical ZnO@TiO_2_ nanowire film. Initially, TiO_2_ NWs were synthesized through hydrothermal approach, where suspension of anatase powders in NaOH aqueous solution was placed in a stainless steel autoclave for reaction at 200°C for 48 h. Sodium titanate precipitate was obtained followed by cation exchange to produce hydrogen titanate NWs, which were further fabricated into TiO_2_ NWs through hydrothermal reaction at 180°C for 3 h [38]. The as-synthesized TiO_2_ NWs were drop-casted on Si substrate to form a homogeneous NW film on the substrate. The substrate was then heated at 200°C to increase NW film adhesion to the substrate. Thereon, a thin ZnO layer of 50 nm was sputtered on top of the substrate as a seed layer for ZnO branches growth. ZnO NWs were grown by hydrothermal method in an aqueous solution containing ZnO precursors (25 mM zinc nitrate hydrate and 25 mM hexamethylenetetramine) at 80°C [39]. Resultant hierarchical micro-nano structure consisting of ZnO NWs branches on TiO_2_ NW backbone (ZnO@TiO_2_ NWs) was obtained after hydrothermal growth for 2 h. Vertical ZnO NW arrays were also synthesized using similar hydrothermal approach as control samples (ZnO NWs), where a 50 nm ZnO thin layer was sputtered on top of Si substrate, followed by hydrothermal growth of ZnO NWs for 2 h.

**Figure 2.**
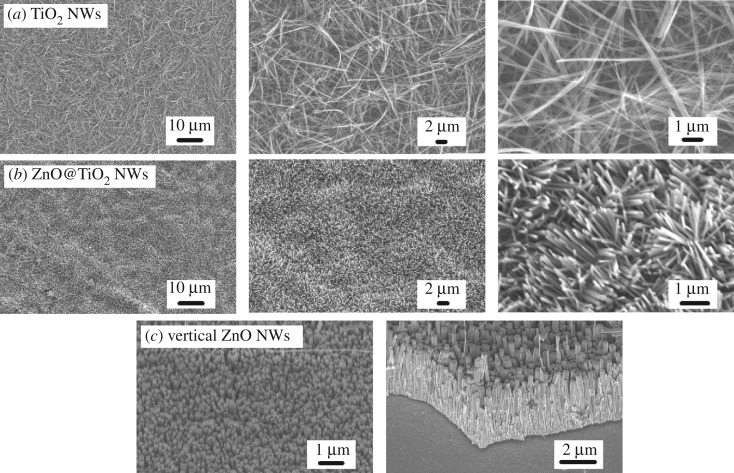
SEM images of (*a*) 1D TiO_2_ nanowire film, (*b*) hierarchical ZnO@TiO_2_ NW film, and (*c*) vertical ZnO NWs. The right sub-panels show SEM images under different magnifications.

Scanning electron microscope (SEM) images of the resultant structures are shown in figure 2. TiO_2_ NWs with diameter of about 100 nm and length more than 10 µm were homogeneously dispersed on Si substrate (figure 2*a*). The NWs were randomly oriented and entangled with each other forming a dense network structure. After hydrothermal growth, ZnO NWs of approximately 90 nm in diameter and 1 µm in length were branched to the TiO_2_ NW backbones. As a result, a denser micro-nanoscale NW structure was obtained as shown in figure 2*b*. Compared to the 1D TiO_2_ nanowire network, micro-nanoscale hierarchical ZnO@TiO_2_ NW structure possessed larger effective surface area, which may be important in amplifying the wetting behaviour according to the Wenzel model, or may entrap air pockets to support liquids according to the Cassie–Baxter model. The as-fabricated hierarchical NW network presented microscale roughness resulting from the underlying TiO_2_ NW backbone as well as nanoscale features from the ZnO nanospikes. The hierarchical network showed a highly homogeneous morphology across the whole surface with few defects. In figure 2*c*, control sample of well-aligned ZnO NW were produced, where the ZnO NWs with diameter of 100 nm and length of 3 µm were grown densely and vertically on substrate surface. In contrast to the hierarchical ZnO@TiO_2_ NW which consisted of both microscale roughness and nanoscale features, the top surface of the densely packed and vertically aligned ZnO NWs only presented nanoscale feature.

**Figure 3.**
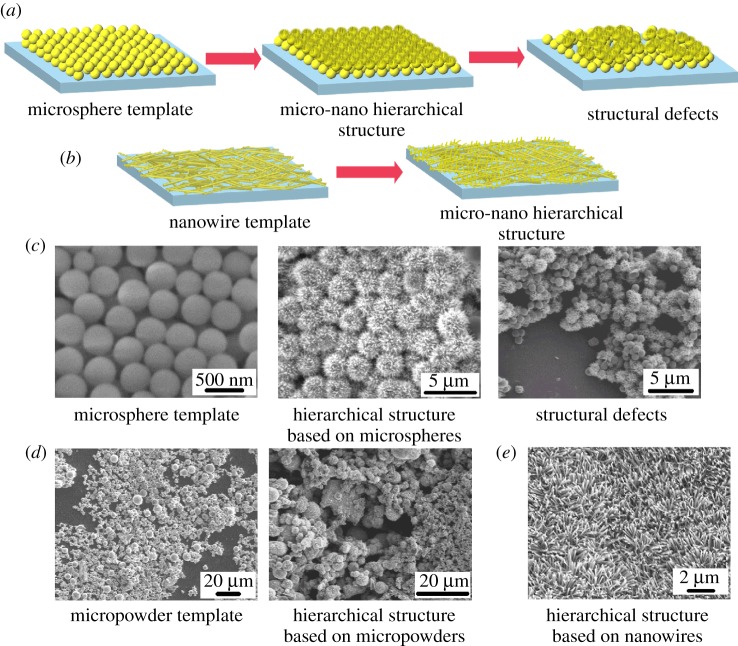
(*a*,*b*) Illustrations of micro-nanoscale hierarchical structure construction using (*a*) microspheres or (*b*) nanowires as micro-scale templates. (*c*) SEM images of microsphere layers prepared via drop-casting, and the micro-nano hierarchical structure fabricated based on the microsphere templates. Large surface defects and a greatly varied surface roughness were observed. (*d*) SEM images of micropowder layers prepared via drop-casting, and the micro-nano hierarchical structure fabricated based on the micropowder templates. Large surface defects and a greatly varied surface roughness were observed. (*e*) Micro-nano hierarchical structure fabricated based on the TiO_2_ NW templates. Homogeneous surface topography and roughness were observed.

Figure 3*a*,*b* illustrates the advantages of using NWs as microscale templates for hierarchical structure fabrication compared to conventional microspheres. The TiO_2_ NWs were in micro-level length and nano-scale diameter. Compared to microspheres, NWs have larger effective area for contacting with the substrate or other NWs, which is favourable for NWs to form a robust network film with smaller variation on surface roughness. This is in contrast to using microspheres or micropowders as microscale templates, where microparticles tended to induce large variations in surface roughness and form defects on the surface. For example, as shown in figure 3*c*, microspheres were stacked on substrate and ZnO nanospikes were fabricated on their surface through hydrothermal reaction. However, microscale defects or vacancies as presented were unavoidable due to imperfect covering of microspheres on the substrate surface. Similar results were also observed for irregular micropowders (figure 3*d*), where uneven layers of micropowders were formed on the substrate. Though ZnO nanospikes could be produced on the micropowder surface, the micro-nanoscale features had large variations among different areas due to the unevenness of the underlying micropowder layers. Instead, by employing TiO_2_ NWs for constructing microscale topography of the underlying layer, the NWs exhibited a relatively homogeneous morphology at different locations. This resulted in a hierarchical NW network with less variation on surface roughness after the TiO_2_ NW backbones were branched by secondary ZnO nanospikes (figure 3*e*).

**Figure 4.**
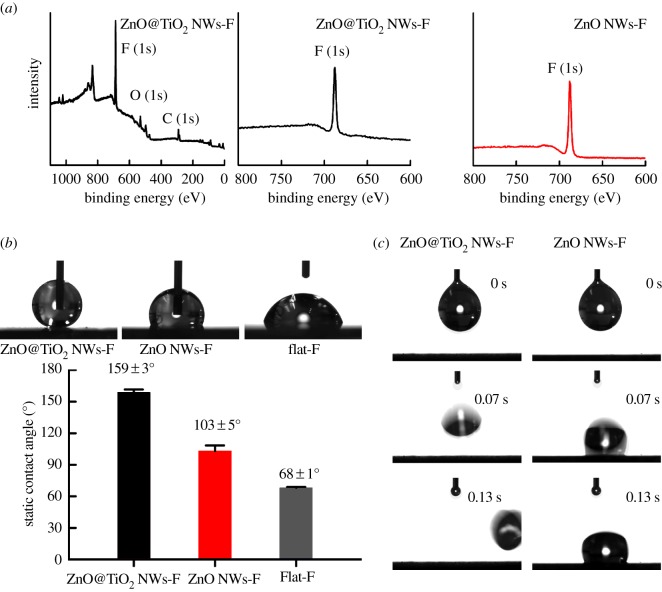
(*a*) XPS spectra of fluorinated hierarchical ZnO@TiO_2_ NWs-F film and vertical ZnO NWs-F. (*b*) Optical images and statistical results of the static contact angles of water on different fluorinated surfaces including ZnO@TiO_2_ NWs-F coating, vertical ZnO NWs-F and fluorinated flat substrate (Flat-F). (*c*) Water repellent test on ZnO@TiO_2_ NWs-F and vertical ZnO NWs-F. Time-resolved images of bouncing experiment for 5 µl water drops on ZnO@TiO_2_ NWs-F and vertical ZnO NWs-F are shown.

To achieve desirable water-repelling property, samples were further modified to be superhydrophobic by conjugating fluorinated silane (perfluorooctyltriethoxysilane [28,34,40]) on the surface to form a chemically bonded low surface energy layer. Briefly, the surface of ZnO@TiO_2_ NWs were coated with perfluorooctyltriethoxysilane using vapour-phase deposition by placing the samples in a vacuum desiccator overnight together with open glass vials containing perfluorooctyltriethoxysilane. After reaction the samples were calcinated at 120°C for 10 min. Similar fluorination process was conducted on control samples including vertical ZnO NWs as well as flat Si substrate. X-ray photoelectron spectroscopy (XPS) was carried out to verify the presence of the fluorinated coating. The pronounced F peaks in figure 4*a* confirmed successful fluorination for both hierarchical ZnO@TiO_2_ NWs and ZnO NWs.

Contact angle (CA) measurements were performed to investigate the wetting properties of hierarchical ZnO@TiO_2_ NW structure after fluorination. Figure 4*b* demonstrates the static CAs of water drops on different samples, including fluorinated ZnO@TiO_2_ NW coating (ZnO@TiO_2_ NWs-F), fluorinated vertical ZnO NWs (ZnO NWs-F), and fluorinated flat Si substrate as controls. The fluorinated hierarchical ZnO/TiO_2_ surface exhibited high CA of 159 ± 3°. On the other hand, the contact angles of control samples were much lower, where the CAs were 103 ± 5° and 68 ± 1° for fluorinated vertical ZnO NWs and flat substrate respectively. Bouncing behaviour of water droplets was further examined on different structured surfaces in order to evaluate the water-repelling property (figure 4*c*). Water droplets could readily bounce off the hierarchical ZnO@TiO_2_ NWs-F surface, indicating the ZnO@TiO_2_ NWs-F coating was highly repellent to water. In contrast, water drops could not bounce on fluorinated vertical ZnO NWs as well as fluorinated substrate surface, suggesting that the presence of micro-nanoscale features was essential for the surface to possess water-repellent property. These results were consistent with previous reports that micro-nanoscale topography significantly contributes to the superhydrophobic performance of surface [36,41,42]. In our case, the coupling of ZnO nanospikes with the underlying NW microtopography greatly increased the hydrophobicity of the surface compared to the ZnO NWs with nanoscale topography alone.

The wetting property of a surface is highly dependent on its surface topography and surface energy. For vertical ZnO NWs-F array, though fluorination substantially reduced the surface energy, it is still insufficient to achieve superhydrophobicity since the water drops could readily penetrate into the grooves between the NWs through capillary action. In contrast, the larger microscale structure provided by TiO_2_ NWs coupled with the ZnO sub-NWs may amplify the superhydrophobic effects according to the Wenzel state, or air is likely to be entrapped more effectively by these nanowire branches according to the Cassie–Baxter model, which may prevent liquid permeation and contribute to the enhanced non-wetting properties. The coexistence of microscale topography and nanoscale feature of ZnO@TiO_2_ NW resulted in different behaviours of water drops impacting on hierarchical ZnO@TiO_2_ NWs-F and vertical ZnO NWs-F surfaces, which was in agreement with previous studies that have shown hierarchical structure was critical for water-repellent properties.

**Figure 5.**
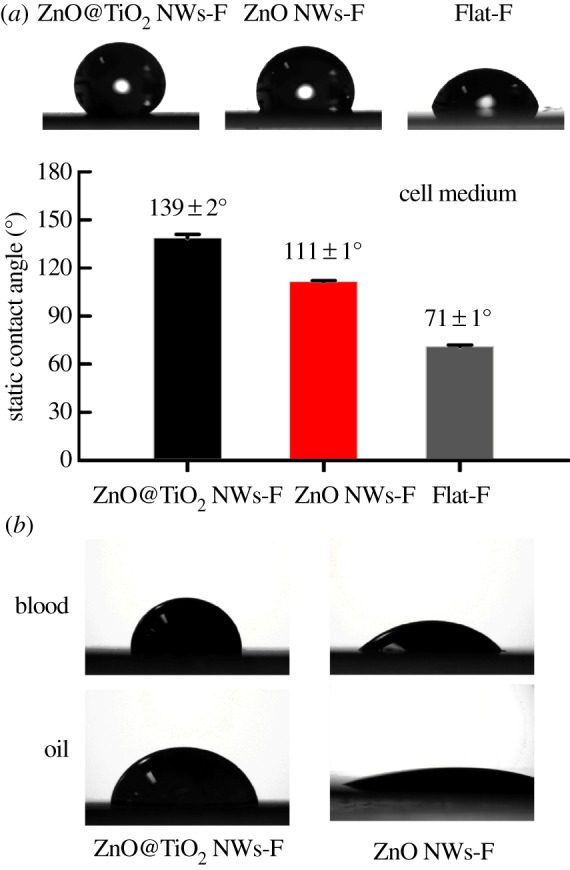
(*a*) Optical images and statistical results of the static contact angles of cell medium on different fluorinated surfaces including hierarchical ZnO@TiO_2_ NWs-F coating, vertical ZnO NWs-F and Flat-F. (*b*) Optical images of blood and oil drops on hierarchical ZnO@TiO_2_ NWs-F film and vertical ZnO NWs-F.

In addition to water, the wetting behaviours of a series of liquids including cell culture medium, blood and corn oil were analysed on the samples (figure 5). Static contact angles of cell medium droplets on fluorinated ZnO@TiO_2_ NW network, fluorinated vertical ZnO NW array and fluorinated flat substrate were measured to be 139 ± 2°, 111 ± 1° and 71 ± 1°, respectively. These results indicate that the ZnO@TiO_2_ NWs-F coating was nearly superhydrophobic to bio-fluids which contain complex molecules such as proteins and other organic molecules. In addition, the contact angles of blood as well as corn oil were tested on the samples. Due to the lower surface tension of blood and oil droplets, these liquids tend to contaminate surface easily and have lower values of contact angle to many existing surfaces. The ZnO@TiO_2_ NWs-F coating displayed contact angles of 89.9 ± 1.3° to blood and 41.2 ± 1.3° to corn oil, which were significantly higher than those on fluorinated ZnO NWs where the contact angles were 41.2 ± 1.3° to blood and 11.7 ± 3.8° to corn oil. Although the surface energy of ZnO@TiO_2_ NWs-F coating was not sufficiently low to repel blood and corn oil, their contact angles were significantly higher than those on fluorinated ZnO NWs, thus confirming the ZnO@TiO_2_ NWs-F coating was much more hydrophobic owing to the co-presence of microscale and nanoscale features.

## 3. Conclusion

In summary, using one-dimensional TiO_2_ NWs as microscale templates, micro-nanoscale hierarchical nanowire network with excellent water-repelling property was successfully developed. TiO_2_ NWs of micrometres length not only provided sufficient contact area with the substrate or NWs entangling for producing a robust network film but also served as excellent backbone structure for ZnO NW branches growth. The hierarchical structure exhibited water-repelling properties after surface modification, displaying excellent superhydrophobicity compared to flat substrate or vertical ZnO NW substrate without hierarchical micro-nanoscale structure. This hierarchical nanowire-based superhydrophobic coating possessed homogeneous morphology in contrast to conventional microparticles/powders-based micro-nanoscale structure where significant surface defects or vacancies were present due to highly varied surface morphologies. Our work provides a promising approach of designing hierarchical nanowire film structure that offers great superhydrophobicity, which is a versatile approach for the coating of a wide range of materials to be self-cleaning.

## Supplementary Material

Experimental methods for fabrication and characterization of TiO_2_ nanowire-templated hierarchical nanowire network
