# Supplementary material for: TiO2 nanowire-templated hierarchical nanowire network as water-repelling coating
Source: R Soc Open Sci. 2017 Dec 20;4(12):171431. doi: 10.1098/rsos.171431 (PMC5750032; doi:10.1098/rsos.171431)
Supplement: Experimental methods for fabrication and characterization of TiO2 nanowire-templated hierarchical nanowire network [file rsos171431supp1.docx]

**Supporting Information for**

**TiO_2_ nanowire-templated hierarchical nanowire network as water-repelling coating**

*Tian Hang,^1,^*^+^  *Hui-Jiuan Chen,^1,+^ Shuai Xiao,^1^ Chengduan Yang,^1^ Meiwan Chen, ^1,2^ Jun Tao,^1^ Han-ping Shieh,^1^ Bo-ru Yang,^1^ Chuan Liu, ^1^ Xi Xie^1,*^*

^1^State Key Laboratory of Optoelectronic Materials and Technologies, School of Electronics and Information Technology; The First Affiliated Hospital of Sun Yat-Sen University; Guangdong Province Key Laboratory of Display Material and Technology, Sun Yat-Sen University, Guangzhou, China

^2^Institute of Chinese Medical Sciences, University of Macau, Avenida da Universidade, Taipa, Macao SAR, China

^3^Department of Photonics and Display Institute, National Chiao Tung University, Hsinchu, Taiwan

**Author Information**

^┼^ These authors contributed equally to this work.

* To whom correspondence may be addressed. Corresponding to: Xi Xie, [xiexi27@mail.sysu.edu.cn](mailto:xiexi27@mail.sysu.edu.cn)

**Experimental Methods**

**TiO_2_ Nanowire (NW) Thin Film Fabrication**

Hydrogen titanate NWs (H_2_Ti_3_O_7_·nH_2_O) were synthesized using a hydrothermal method in an alkaline environment. First, 0.1 g of anatase powder (SigmaAldrich, USA) was dispersed in 20 ml of a 10 M NaOH aqueous solution. The solution was stirred at room temperature for 1 h to ensure the homogeneity. Then the suspension was transferred into a Teflon-lined stainless steel autoclave at 200 ^o^C for 48 h. After the hydrothermal treatment, the suspension was separated by centrifugation. The obtained white sodium titanate (Na_2_Ti_3_O_7_) precipitate was washed, dried and then soaked in a 0.1M HCl aqueous solution for a complete cation exchange between the sodium ions and the hydrogen ions for 24 h at room temperature. Hydrogen titanate nanowires were obtained after the suspension was washed with deionized water several times. Finally, the anatase TiO_2_ nanowires were fabricated from the as-prepared hydrogen titanate nanowires using a low-temperature synthesis method through a hydrothermal treatment with deionized water at 180 ^o^C for 3 h along with centrifugation, washing and filtration steps. The obtained TiO_2_ nanowires were drop-casted on Si substrate and heated at 200 ^o^C for 10 min.

**Fabrication of Microparticles Thin Film**

TiO_2_ microspheres (~800 nm, Microspheres & Nanospheres) or glass micropowders (~1 μm, Cospheric) were dispersed in ethanol. The particle solution was drop-casted onto a glass substrate (cleaned with O_2_ plasma) and dried. The substrate was placed in atomic layer deposition (Cambridge Nanotech) system. The substrate along with the particles were coated with 20 nm ZnO layer using diethylzinc/H_2_O (both 0.015 s pulse time, and 8 s waiting time) process (200 pulse cycles at 200 ^o^C).

**Growth of ZnO Nanospikes**

Branched ZnO nanowires were synthesized through hydrothermal method. A thin ZnO layer of 50 nm was sputtered on TiO_2_ nanowire film surface as a seed layer, then incubated in aqueous solution containing 25 mM zinc nitrate hydrate [Zn(NO_3_)_2_•6H_2_O, Sigma-Aldrich] and 25 mM hexamethylenetetramine (C_6_H_12_N_4_, HMTA, Sigma-Aldrich) for 2 h at 80 ^o^C. The obtained samples were rinsed with DI-water for five times.

**Synthesis of Vertical ZnO Nanowires**

Si wafer was sputtered with a thin ZnO layer of 50 nm. Then the wafer was incubated in aqueous solution containing 25 mM Zn(NO_3_)_2_•6H_2_O and 25 mM HMTA at 80 ^o^C for 2 h. After reaction, the substrate was rinsed with DI-water five times.

**Fluorinated Functionalization**

The hierarchical nanowire film and vertical ZnO NW samples were placed in a vacuum desiccator overnight together with open glass vials containing anhydrous heptane solution containing 2 wt% perfluorooctyltriethoxysilane (C_14_H_19_F_13_O_3_Si, Sigma-Aldrich) for 12 h. After that, the samples were rinsed with heptane and acetone for 3 times, and then calcinated at 120 ^o^C for 10 min.

**Characterization**

The morphology of the samples was characterized with a field-emission scanning electron microscope (Zeiss). X-ray photoelectron spectroscopy (XPS, PHI 5000) measurement was conducted to analyze the surface composition.

**Static Contact Angle Analysis**

Contact angles were measured with Goniometer measuring system to characterize the wetting properties. Deionized water and various liquids including cell culture medium (Thermo Fisher Scientific), blood (from C57BL/6 mice) and corn oil (Sigma-Aldrich) were tested as probe liquids. Bouncing behavior was recorded by free falling of 5 μl water drops on different surfaces.

**Abbreviations**

NWs, Nanowires

SEM, Scanning electron microscope

XPS, X-ray photoelectron spectroscopy

CA, Contact angle

| **Abbreviation** | **Structure** | **Description** | **Contact angle (SD) (^o^)** | | | |
| --- | --- | --- | --- | --- | --- | --- |
|  |  |  | **Water** | **Cell medium** | **Blood** | **Oil** |
| ZnO NWs | **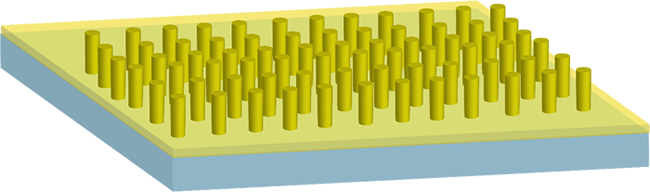** | Vertical ZnO nanowires | - | - | - | - |
| ZnO@TiO_2_ NWs | **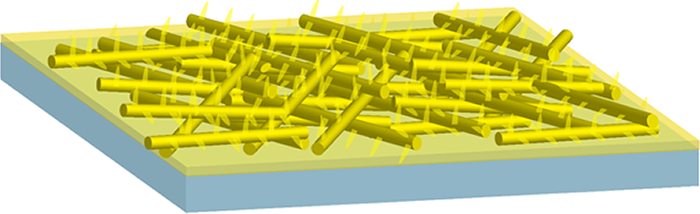** | Hierarchical nanowire film: TiO_2_ nanowires with ZnO nanowire branches | - | - | - | - |
| Flat-F | 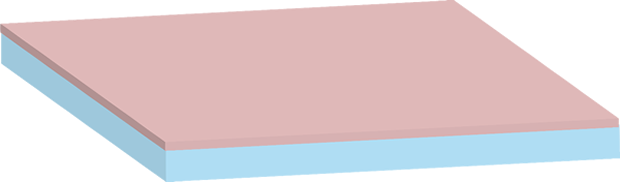 | Fluorinated flat substrate | 68.1 (0.8) | 71.0 (0.9) | - | - |
| ZnO NWs-F | 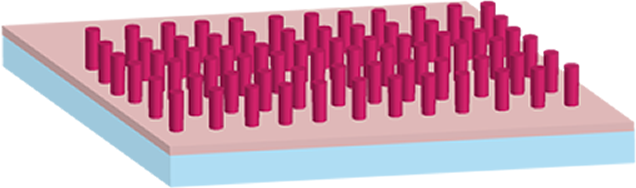 | Fluorinated vertical ZnO nanowires | 103.0 (5.3) | 111.3 (0.7) | 41.2 (1.3) | 11.7 (3.8) |
| ZnO@TiO_2_ NWs-F | 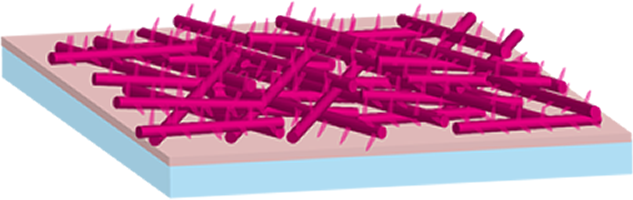 | Fluorinated hierarchical ZnO@TiO_2_ nanowire film | 158.9 (2.6) | 138.7 (2.2) | 89.9 (1.3) | 78.3 (1.4) |
